# Rayada specialty: the forgotten resource of elite features of rice

**DOI:** 10.1186/1939-8433-6-41

**Published:** 2013-12-21

**Authors:** A N M Rubaiyath Bin Rahman, Jianhua Zhang

**Affiliations:** 1 Department of Biology, Hong Kong Baptist University, Hong Kong, China; 2 School of Life Sciences and State Key Laboratory of Agrobiotechnology, The Chinese University of Hong Kong, Shatin, Hong Kong, China

**Keywords:** Rayada rice, Deepwater rice, Stress tolerance, Rice genetics, Rice breeding

## Abstract

Crop domestication and thereafter gradual selection or directional breeding have narrowed the genetic diversity of elite varieties and even promoted gathering of deleterious mutations in their stress response mechanisms, whereas local ecotypes, landraces and wild relatives still growing on native environment and preferences keep the genetic diversities for features like stress tolerance. Rayada is such an exceptional ecotype, variant of typical deepwater rice, completely endemic to certain areas of Madhumati river tracts of Bangladesh and still shares some features of wild rices. Multiple physiological features of Rayadas are distinctly different from typical deepwater rice. Tolerance to prolonged flood, submergence and cold are special features along with strong photoperiod sensitivity and lack of dormancy. Moreover, longer root system and prompt recovery capacity make it as an elite resource of stress tolerance. However, it has long been neglected because of mainly its long life cycle and poor yield. This review examines the specialty of Rayada rice and the potential use of its unique traits.

## Introduction

Being sessile organisms, plants have evolved with special capacity to sense, respond and adapt to unfavourable environmental conditions. Water stresses, either excess (flooding) or shortage (drought) of water, significantly limit plant growth and development and obviously crop production. The natural capacity of these stress tolerance mainly depends on plants genome specialty and efficiency of activation of adaptive mechanisms (Chinnusamy et al. 2004). Moreover, genetic diversity is the raw materials of plant breeding as well as crop improvement programme. However, crop domestication and thereafter gradual selection or directional breeding have narrowed the genetic diversity of elite varieties, even promoted gathering of deleterious mutations in their stress response mechanisms (Tang et al. 2010), whereas local ecotypes, landraces and wild relatives growing on native environment and preferences still keep genetic diversities for features like stress tolerance, disease resistance etc. (Suslow et al. 2002). Rayada is such an exceptional ecotype, variant of typical deepwater rice, completely endemic to Bangladesh and still shares some features of wild rices. This review focuses on specialty of Rayada rice, elaborated their physiological and other agronomic features that are distinctly different from typical deepwater rice.

## Review

### Bangladesh: Hub of deepwater rice

Flood and rice are two indispensable features of Bangladesh landscape, one of the most flood prone countries in the world. Most areas of this country is less than 5 meters above sea level and hence almost every year, more than 20% areas becomes inundated with an additional 42% with risk of inundation of varied intensity (Ahmed and Mirza 2000; Dasgupta et al. 2011). Apart flood, due to its wide variation in land, topography and seasons, genetic diversity of rice of Bangladesh is highly enriched, especially for deepwater rice. A nationwide survey identified 3820 varieties names of deepwater rice in Bangladesh and a total of 659 deepwater cultivars were collected, categorized and stored in both BRRI and IRRI gene bank (Bashar et al. 2004). Deepwater rice, locally regarded as Broadcast aman, is one of the special rices cultivated in Bangladesh since time immemorial. Deepwater rice is defined as rice cultivated in flood plain deeper than 50 cm for one month or longer during the growing season (Catling et al. 1988; Catling 1992).

**Figure 1:**
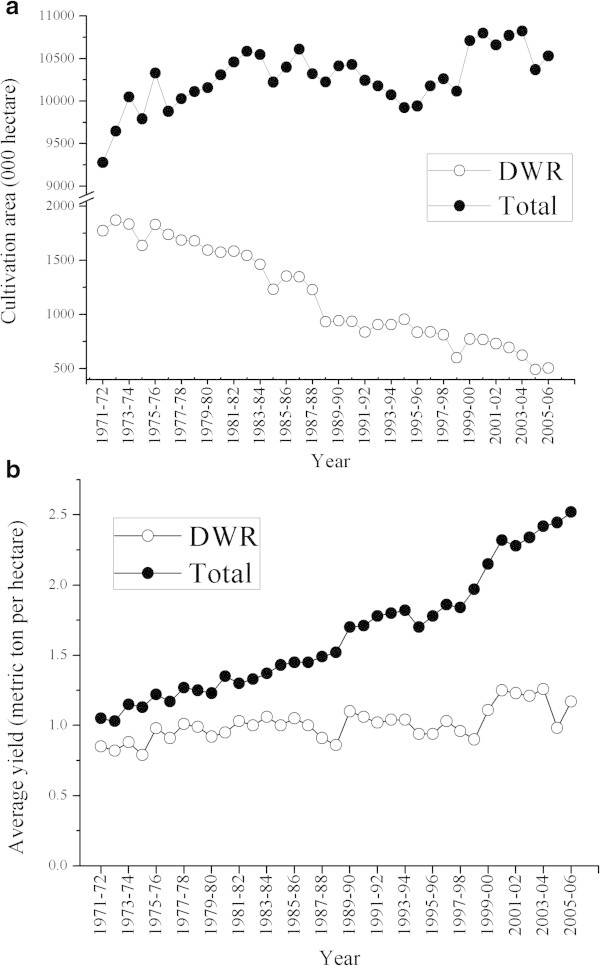
**Status of deepwater rice cultivation in Bangladesh.** Comparative scenario of cultivation area **(a)** and average yield **(b)** of deepwater (DWR) and total rice cultivation in Bangladesh from 1971–72 to 2005–06.

In 1971–72, deepwater rice was cultivated in almost one fifth area (18.14%) of the total rice cultivated area of Bangladesh. However, in 2005–06, it was reduced to 505.46 thousand hectare area, i.e. 4.08% of total rice cultivation area (Figure 1a). The gradual decreasing trends of deepwater rice cultivation over the past 34 years was due to the increased cultivation of high yielding modern varieties with irrigation facilities in boro season since the yield of deepwater rice is generally low. The yield of deepwater rice over this period has always been around 1 ton/hectare (Figure 1b), implying the absence of varietal development, whereas yields of other rice types have increased gradually due to both improved modern varieties and agricultural practices.

There are three major types of deepwater rice cultivated in Bangladesh. They are: 1) Typical deepwater rice, 2) Bhadoia/Ashwina and 3) Rayada (Catling 1992; Bashar et al. 2004). All of them are collectively considered as deepwater rice having similarity in elongation capacity after rising flood water level, kernel colour and yield, although there are some distinct differences among them, especially to Rayada rice. Table 1 shows the key differences among them comparing with wild rice, *Oryza rufipogon*. Ashwina/Bhadoia are almost similar to typical deepwater rice except less or no sensitivity to photoperiod (Bashar et al. 2004) and they usually flower in Bengali calendar months of Bhadra/Ashwin (August/September), hence coined the term Bhadoia/Ashwina (Haque 1974). Among these three types, Rayadas are most primitive, still sharing some features of wild rices (Khush 1997).

**Table 1:** Comparative features of different types of deepwater and wild rice of Bangladesh

**Features**	**Typical deepwater rice**	**Ashwina/Bhadoia**	**Rayada rice**	**Wild rice (*O. rufipogon *)**	**References**
Elongation ability	Yes	Yes	Yes (faster)	Yes (faster)	Perez and Nasiruddin ( 1974); Hasanuzzaman ( 1974); Catling ( 1992); Vaughan ( 1994); Khush ( 1997); Bashar et al. ( 2004); Baruah et al. ( 2009)
Life cycle (months)	6-8	6-8	12 and 6-8	Perennial	
Cold tolerance	No	No	Yes	Varying degree of tolerance	
Photoperiod sensitivity	Varying degree of sensitivity	Less or no	Strongly sensitive	Sensitive	
Seed dormancy	High	High	No	High	
Responsiveness of short day in early vegetative stage	Yes	Yes	No	(Not known)	
Distribution	South and Southeast Asia	Bangladesh and India	Endemic to Bangladesh	South and Southeast Asia, Latin America, Australia, West- Africa	
Examples	Habigonj Aman I -VIII	Ashwina	Kaladigha, Beto, etc.	Jhoradhan	

### Rayada: endemic deepwater rice ecotype

Rayada is unique rice ecotype, distinctive group of deepwater rice, totally endemic to certain area of Bangladesh and have multiple physiological features distinctly different from typical deepwater rice (Table 1) (Perez and Nasiruddin 1974; Catling 1992; Bashar et al. 2004). They are photoperiod insensitive in early vegetative stage but strongly sensitive for flowering in late vegetative stage (Vergara and Chang 1985). Moreover, they completely lack seed dormancy, typically different to other deepwater rices along with longer life cycle of 12 months. However, there are two types of Rayada rice based on the duration of their life cycle i.e. (1) 12 months Rayada and (2) 6–8 months Rayada (Perez and Nasiruddin 1974; Bashar et al. 2004). Rayada rice was also described as a distinct group in very early literature (Watt 1891). Some biochemical studies also identified and supported its separate status to deepwater rice (Glaszmann 1987). Rayada rice was ranked a separate group (Group-IV) from typical deepwater rice varieties (Group-III) based on fifteen polymorphic loci coding for 8 enzymes on 1688 traditional Asian varieties.

**Figure 2:**
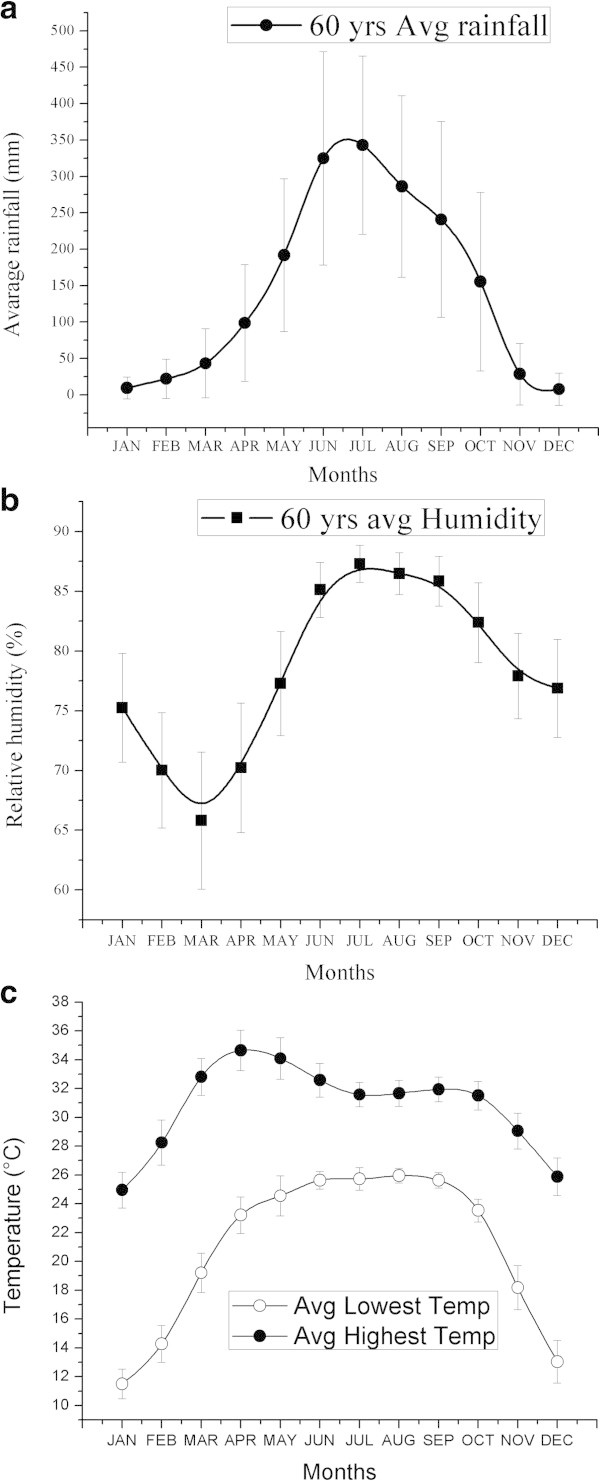
**Climatic features of Rayada growing areas. a)** average rainfall, **b)** humidity and **c)** lowest and highest temperature. Long term (1948–2008) average climatic status of Rayada growing area was calculated based on data of two nearby weather stations (Faridpur and Jessore) of Rayada grown area. Metrological data were collected from Bangladesh Agricultural Research Council website ( http://www.barc.gov.bd/data_stat.php).

The Rayada rice is only cultivated in certain areas of natural lowland (locally called beel) beside the Madhumati river. The Rayada growing area is estimated at 5,180 hectares bordered by Sreepur, Magura (North) to Mollahat, Bagerhat (South), Magura and Narial (West) to Gopalgonj (East) (Perez and Nasiruddin 1974). However, the 12-month Rayada is only cultivated at Kalia in Narail, Mollahat in Bagerhat, and Gopalganj (Perez and Nasiruddin 1974; Bashar et al. 2004) whereas, deepwater rice is cultivated throughout the country except three hill tract districts. Deepwater rice cultivation areas can be divided into mainly two zones. Zone-1 includes greater Faridpur, Jessore, Kushtia, Rajshahi and Pabna districts whereas zone-2 encompasses greater Sylhet, Comilla, Tangail, Dhaka, Noakhali and Mymensingh districts (Ahmed 1974). Soil properties of these two zones are generally different i.e. zone-1 characteristically natural to alkaline, rich in calcium and phosphorus but deficient of nitrogen and potassium. Oppositely, soil of another group is distinctly acidic, poor in calcium and phosphorus and comparatively rich in nitrogen and potassium (Ahmed 1974). Interestingly, Rayadas are only available in zone-1. We have also studied the long term (1948–2008) climatic features (month-wise average rainfall, humidity, and temperature) of Rayada growing area, illustrated in Figure 2.

### Differential traits of Rayada rice

Numerous morphological and physiological differences exist among rice varieties (Chang and Bardenas 1965). For instance, some varieties mature within 80 days whereas Rayadas need a year to complete its life cycle (Khush 1997). Rayadas are cultivated rice of longest life cycle in Bangladesh. It is traditionally cultivated as mixed crop with boro in November/December. Boro is harvested in April/May before onset of flood while Rayada keeps growing and is harvested in the next November/December (Perez and Nasiruddin 1974; Bashar et al. 2004). So from seedling to harvest is around 12 months or a year. Water level increases in around June, peaks in early August and recedes by the end of September or early October. Rayada usually flowers after flood water recedes in late September (Hasanuzzaman 1974), at that time day length already becomes shortened to induce flowering. Rayada type varieties might be eliminated because of the long life cycle (Vergara and Chang 1985).

**Figure 3:**
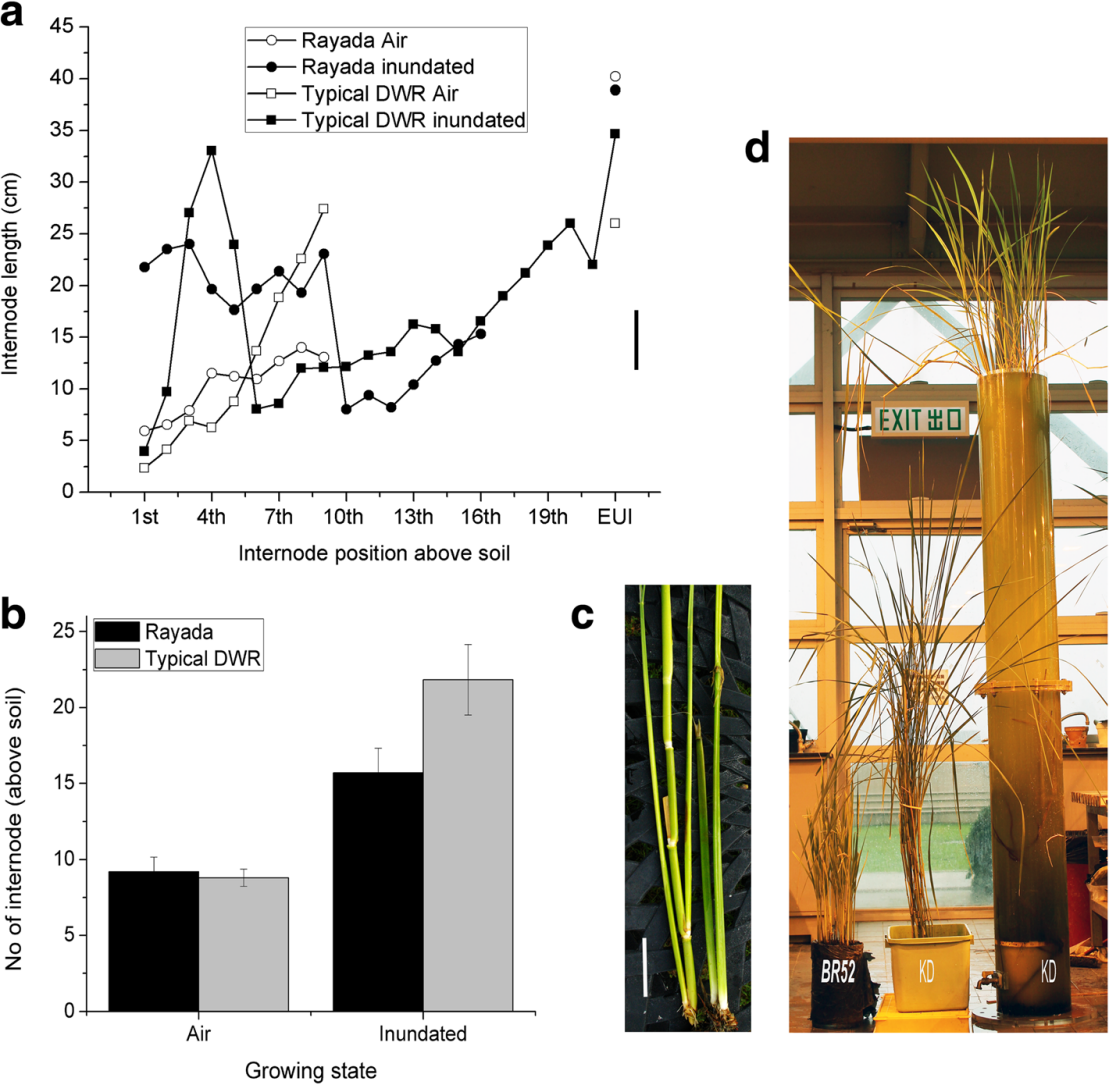
**Internode elongation pattern of Rayada variety, Kaladigha to typical deepwater rice and comparative phenotypes of Rayada after inundation. (a)** internode elongation pattern Rayada and typical deepwater rice (Habigonj amam I-VIII after Hasanuzzaman ( 1974) (bar = SD), **(b)** number of internodes per plants, **(c)** phenotypic comparison of basal internodes (above soil) in Rayada in control and inundated condition (bar = 5 cm), **(d)** comparative phenotypes BRRI52, Kaladigha, and inundated Kaladigha (left to right, respectively) at plants maturity. Plants (4.5 months old) were grown in water tube with increasing water level keeping top leaves above water for 45 days reaching 2.5 meter. Then the plants were kept under around 2.5 meter inundation (water renewal fortnightly) for rest of months (around 7 months) till seed mature. Basal internodes/nodes (3–4) beneath the soil were excluded in estimation and comparison.

It is evident that deepwater rice elongates its internode after flooding as a survival strategy (Keith et al. 1986). However, the pattern of internode elongation of Rayadas is not clearly evident. In our experiments, interestingly, we observed all internodes under water were significantly elongated after prolonged inundation (Figure 3a) and plants became more than 3 meter tall (Figure 3d). Artificial inundation induced internode elongation of 2–4 times longer than control (Figure 3a, c). Notably, basal internode above soil was found equally elongated to middle internodes and significantly different from control plants (Figure 3c). We also compared internode elongation patterns of Rayada variety, Kaladigha with typical deepwater rice (Habigonj aman I-VIII) reported earlier (Hasanuzzaman 1974). Air grown plants of both types showed almost similar pattern of internode length except much elongated distal internodes in typical deepwater rice (Figure 3a). However, basal internode elongation pattern in Rayada is significantly different from that of deepwater rice. Some middle internodes of typical deepwater rice became similar to air grown plants (Figure 3a). In addition, numbers of nodes were found significantly increased after inundation in both types, deepwater varieties showed more internodes than Rayada because of natural flood grown condition whereas Rayada variety was grown in greenhouse in 2.5 meter water tube (Figure 3a, b). Internodes above the water level became same as control but developed nodal tillers. However, highest elongated internode was the elongated uppermost internode (EUI) both in control and inundated plants (Figure 3a). The *eui* mutant was reported to accumulate exceptionally large amounts of biologically active gibberellins (GAs) in the uppermost internode (Zhu et al. 2006).

Control of tolerance to complete and partial submergence are regulated by similar type ERF (ethylene response factor) transcription factors by opposite mechanisms. Under complete submergence, SUB1 restricts GA responsiveness by enhanced SLR1 expression (Xu et al. 2006; Bailey-Serres et al. 2010) whereas in partial submergence, SK genes enhance GA responsiveness by some unknown mechanisms (Hattori et al. 2009). Some *aus* ecotypes such as FR13A has SUB1A gene whereas deepwater even wild rice species possess SK genes for internode elongation (Hattori et al. 2009). Rayada rice is efficiently able to elongate its internodes after partial submergence (Figure 3). Similarly, in our experiments we found it has equal capacity to withstand complete submergence of 2 weeks with similar performance to Sub1A introgressed rice (BRRI 52) (data not shown). Similar observation of complete submergence tolerance was also previously reported in Rayada rice (Perez and Nasiruddin 1974). Complete submergence tolerance of Rayada rice might be regulated by SUB1A or some other means. However, two Sub1A like alleles (*OrSub1A-1, OrSub1A-2*) have already been reported in wild rice *O. rufipogon* (Li et al. 2011). So it is more likely that Sub1A type submergence tolerance regulates complete submergence in Rayada rice.

Prolonged seed dormancy is one of the distinctive features of the deepwater rices (Beachell 1974). Deepwater rice cultivars have a dormancy of 25–56 days (Catling 1992). Moreover, most wild rices also have strong seed dormancy (Cai and Morishima 2000). Dormancy is essential for deepwater rice to prevent inadvertent germination prior to harvesting of mature seeds while still on the mother plant under high humid or wet even inundated conditions. Among deepwater rices, Rayada is the only exception, having no seed dormancy. Rayada seeds are sown within weeks, even days after harvest (Catling 1992). We also reexamined this feature and found efficient seed germination capacity even after fresh harvest (data not shown). Lack of seed dormancy seems an adaptive feature of Rayada rice because of its yearlong life cycle, highly photosensitive nature and flowering time along with climatic features. During seed maturation period (late October-November), the rainfall, humidity and temperature, drop to lower extend to avoid unexpected germination (Figure 2a, b, c).

Generally japonica rice is considered more cold tolerant than the indica (Andaya and Mackill 2003). Among indicas, cold tolerance is another important feature of Rayada rice and distinctive feature from typical deep water rice (Catling 1992). Rayadas are tolerant to chilling temperature of 10°C. In the winter months the lowest temperature drops to around 10°C in the Rayada growing areas (Figure 2c) and the plants across whole winter months during its initial vegetative growth stages. Typical deep water rice is usually planted in April-May, after the temperature rises (Figure 2c) whereas Rayadas in November-December (Perez and Nasiruddin 1974; Bashar et al. 2004). We also re-examined the cold tolerance of Rayada by incubating in chilling temperature of 10°C for 4 days which resulted *aus* cultivars to cold injury whereas Rayada varieties showed no effect at all (data not shown).

Longer root system is unanimously considered as the feature of drought adaptation or tolerance (Blum 2011). Upland rice usually possesses longer root system and is hence adapted to drought prone area. Single nucleotide polymorphisms (SNPs) among 20 diverse rice varieties and landraces (OryzaSNP panel) including Rayada have been identified for deep exploration of rice diversity and gene–trait relationships and eventually for future rice improvement (McNally et al. 2009). Henry et al. ( 2011) evaluated variation in root system architecture and drought response among these 20 diverse rice genotypes of OryzaSNP panel and identified the *aus* isozyme group, particularly Dular genotype having highest drought resistance by deeper root growth with highest drought response index. Similarly, Gowda et al. ( 2012) examined root growth, water uptake and shoot growth among these 20 OryzaSNP panel under both well-watered and water-stressed conditions. We compared root growth pattern of Rayada to Dular genotype from their results (Gowda et al. 2012). After analysing their data, we identified longer root system of Rayada, even better than Dular genotype both in well-watered and water-stressed conditions. In well-watered condition, Rayada showed root length density (RLD) of 12.87 cmcm^-3^ in surface to 30 cm soil profile, more than 5 times higher than Dular (2.50 cmcm^-3^). Interestingly, in water-stressed condition from surface to 100 cm depth, RLD of Rayada was always higher than Dular, especially in 30–45 cm soil profile, more than doubled in Rayada (RLD 3.77 cmcm^-3^) than that of Dular (1.60 cmcm^-3^). However, inappropriately, Gowda et al. ( 2012) generalized Rayada as *aus* group although they are distinctly different in all aspects of growth and development. We have also re-examined root growth capacity after seed germination and seedling establishment stage among Rayada, typical deepwater rice, flood tolerant and *aus* varieties. Although variation existed among ecotypes/varieties but we observed that Rayadas always displayed longer root systems. Some upland varieties also showed elevated root growth initially but Rayada varieties superseded that due to higher growth rate (data not shown). Flash flood tolerant Sub1A varieties (BRRI51and 52) showed least growth for both root and shoot. Month old seedlings showed distinct differences in phenotypes, especially in root length in Rayada variety (data not shown).

Life cycle of rice consists of three phases: 1) vegetative growth stage, from germination to panicle initiation, 2) reproductive stage, from panicle initiation to flowering (generally 35 days), and 3) the ripening stage, from flowering to ripen grain (usually 30–35 days, could be even 2 months depends on some factors such as water, nutrient richness) (Vergara and Chang 1985). So the duration of the life cycle of rice varieties mainly depends on the vegetative growth phase. Based on photoperiod sensitivity, the vegetative growth phase can be again divided into two phases: a) basic vegetative phase (BVP)-juvenile growth stage, independent to photoperiod and b) photoperiod sensitive phase (PSP). Rice varieties are classified into four types based on photoperiodic sensitivity: 1) Type A- both BVP and PSP are short, such as most modern varieties, 2) Type B - long BVP but short PSP, such as Habigonj Boro 5, BVP and PSP are 57 and 5 days, respectively, 3) Type C– short BVP and long PSP, such as typical deepwater rice varieties and 4) Type D- both long BVP and PSP. Rayadas are the only group of rice categorized type D (Vergara and Chang 1985).

Most of the previous literatures reported BVP range from 10 to 63 days (Gomosta and Vergara 1983). In typical deepwater rice varieties, BVP range from 3 to 43 days whereas that of Rayadas is 70 to 74 days, longest among all rice varieties reported earlier (Vergara and Chang 1985). Longest BVP is the characteristic feature of Rayada makes it non-responsive in early growing stage to short photoperiod in February and early March (Vergara and Chang 1985). Possibly it is because of low temperature in winter months (Nov to Jan) that extended BVP (Gomosta and Vergara 1983). The range of PSP in photoperiod insensitive varieties is 0 to 30 days whereas PSP of photoperiod sensitive cultivars are above 31 days to years (Vergara and Chang 1985). Continuous long photoperiods resulted some cultivars to keep in vegetative stage more than 12 years (Kondo et al. 1942). So experimental duration for PSP determination was usually concluded after 200 days. All of the Rayada varieties tested earlier showed highest limit of PSP, same as typical deep water rice varieties (Vergara and Chang 1985). Critical photoperiod of Rayadas is around 12 hrs. Moreover, photoinductive cycle is also prolonged in Rayadas, more than 2 weeks (Vergara and Chang 1985). Interestingly, most of the wild rices also showed strong photosensitivity (Oka and Chang 1960).

Water stress recovery is crucial for Rayadas as it may be totally lost or damaged if it fails to recover from drought stress before onset of flood. Previous screening of IRRI in the 1981 identified 4 Bangladeshi varieties (Sarsari, Bhabani, Hijaldigha and Tilbazal) as promising deepwater varieties for drought tolerance (Datta et al. 1982). Interestingly, Sarsari showed a similar recovery score as that of two drought tolerant check, Salumpikit and IR442-2-58 (Datta et al. 1982). In our study, we compared Rayada variety, Kaladigha with aus genotype, Dudsor. Kaladigha showed leaf rolling in just minutes after transferring high humidity (90%) growing plants to low humidity (60%) condition (data not shown). High humidity induces ABA 8′-hydroxylase to regulate local and systemic ABA responses in Arabidopsis (Okamoto et al. 2009). So we compared how quickly Rayada and Dudsor respond to recovery after watering. Leaf rolling is a useful indicator of leaf water potential in rice (O'Toole and Cruz 1980). Hence we compared water stress recovery rate by measuring the leaf derolling rate after watering. After analysing the time course photographs, it revealed that Rayada variety sensed watering in just minute and 50% derolled leaves (mostly young leaves) within 4–5 minutes whereas aus genotype sensed and recovered significantly slower than Rayada variety (data not shown). Excellent performance of recovery of Rayada rice here suggests as it could be used as promising materials for future studies of water stress recovery and stress tolerance.

### Origin and evolution of Rayada

The origin and evolution of rice is little bit of a puzzle with considerable debate mainly on single or multiple ancestries with domestication place (Londo et al. 2006; Molina et al. 2011; Huang et al. 2012; Sang and Ge 2013). Most of the scientists believe *O. rufipogon* as progenitor of deepwater rice because it has still deepwater types, adapted to deepwater habitat and having photoperiod sensitivity too (Glaszmann 1987; Catling 1992; Bashar et al. 2004). As elongation ability after flooding is the main feature of both Rayada and typical deepwater rice, hence the evolutionary study of key genes responsible for internode elongation after flooding is also relevant to Rayada. Snorkel genes (SK1 and SK2) allow rice to adapt in deepwater condition by enhancing GA responsiveness (Hattori et al. 2009). Both of the wild species (*O. rufipogon* and *O. nivara*) possess complete SK1, but SK2 of *O. nivara* is truncated due to insertion of transposon. However, *O. glumaepatula*, another wild rice species, have SK2 and SK2-like genes, but deficient of SK1. The phenotypic effect of SK2 is more prominent and wild rice species (*O. rufipogon* and *O. glumaepatula*) possess the SK2 genes, suggesting these genes may have been evolved before or during wild rice speciation (Hattori et al. 2009). Generally, aquatic adaptation is considered as a primitive feature in the context of evolutionary series. However, deepwater adaptation of rice is not a newly evolved adaptation; rather a remnant from evolutionary progenitor (Bashar et al. 2004). Thus the hypothesis of evolution of non-deepwater rice forms through deepwater varieties (Bashar et al. 2004), especially from Rayadas (since being most primitive among deepwater rices), cannot be ruled out unless scientifically proved.

Isozyme study of 8 enzymes using 1688 traditional Asian varieties, however, revealed that Rayadas neither fall under japonica nor indica type, even different than typical deepwater rice thus categorized into separate group (Group-IV) (Glaszmann 1987). Interestingly, Bangladeshi deepwater rice varieties (all three types) were found to possess special isozyme alleles, *Est10-4* and *Amp5-4* which are frequently found in Asian common wild rice *O. rufipogon* but rare in other cultivars (Cai and Morishima 2000). These findings suggest Bangladeshi deepwater rice has also inherited these alleles from its wild progenitor. Generally, it has been considered that deepwater rice varieties belong to indica spp. only, although a very few reports on isozyme analysis showed some of the floating rice of Bangladesh including Rayadas displayed japonica relevance (Cai and Morishima 2000; Wang et al. 2013). However, none of the 188 deepwater rice tested from Thailand, Vietnam and Cambodia showed japonica pattern (Catling 1992). Analysing the polymorphism of nuclear, mitochondria and chloroplast DNA of 193 genotypes, Sun et al. ( 2002) identified japonica type nuclear and chloroplast DNA but indica type mitochondria DNA in some Bangladeshi deepwater landraces including Rayadas. Since some of Bangladeshi deepwater rice varieties including Rayada showed japonica relevance as well as japonica type nuclear and chloroplast DNA, so considering other features of Rayadas altogether (such as endemic nature, yearlong life cycle*,* strong photoperiod sensitivity, multiple stress tolerances and elongation ability after flooding that are resemble to wild rice), collectively might deduce Rayadas as a possible missing link between indica-japonica differentiation. However, most of the large scale molecular studies (GWAS etc.) of rice omitted Bangladeshi unique varieties such as Rayadas as experiment material hence the enigma lingers.

### Rayada rice: the forgotten resource

Although Rayadas possess several desired features of abiotic stress tolerances, but it was always been neglected mainly because of the yearlong life cycle, poor yield and strong photoperiod sensitivity nature. Moreover, endemic nature of Rayadas is a reason to distract the focus of its varietal improvement as only 10% area of deepwater rice cultivation of Bangladesh was of Rayadas. So, all the improved deepwater varieties developed and released since 1920–21 (after the establishment of Dhaka and Habigonj station) either through pure line selection or breeding methods of the country were confined to typical deepwater type (Zaman 1977; Bashar et al. 2004). Similar pattern of less attention to Rayadas was also observed in the IRRI breeding programme. Among first 50,000 cross made by IRRI, only 15 of them (cross no IR16291 to IR16304) directly used Rayada as parents (IRRI 1985). Moreover, even IRRI has left deepwater rice variety improvement program out of its priority list, similar situation also exists in Bangladesh Rice Research Institute. Fortunately, OryzaSNP panel included Rayada as a diverse genotype (McNally et al. 2009), thus might help the gene mining of this special ecotype.

### Potential uses of Rayada traits

Rayadas could be potential resources of abiotic stress tolerance traits like flood, cold and drought (Perez and Nasiruddin 1974; Glaszmann 1987; Bashar et al. 2004) as it still shares some features of wild rice (Khush 1997). In addition, it displayed longer root system both in well-water and water-stressed conditions, even better than best performing aus genotype, Dular among 20 OryzaSNP panel (Gowda et al. 2012). Moreover, in our study we also observed quicker recovery and stomatal responses (data not shown) etc. along with longer root system in Rayada rice. Elongation ability after flooding as well as faster recovery and quicker stomatal responses of Rayadas suggest it might possess efficient water sensing mechanism. Moreover, all of these three features are considered as traits of drought tolerance or adaptation; interestingly, to some extent they are present in some varieties of Rayadas. Stress tolerance features of Rayadas are not only limited to abiotic stresses but it also possesses some biotic stress tolerance features like ufra disease resistance, as reported earlier (Catling et al. 1988a).

Interestingly, both partial and complete submergence tolerance capacities that we observed in our study, direct Rayadas rice not only special but also unique. Similar feature of dual submergence tolerance capacity was also reported some 40 years ago but still scientifically unexplored (Perez and Nasiruddin 1974). It would be fascinating to identify how Rayadas sense, differentiate and activate signalling pathways after such stresses.

Functional role of snorkel genes in deepwater rice has been already revealed (Hattori et al. 2009) although how SK genes regulate GA responsiveness is still unknown. Moreover, several other minor QTLs (qTIL2 and qTIL4) identified in deepwater rice (Nagai et al. 2012) also need to be characterized genetically. Moreover, focused study on Rayada or deepwater rice would enrich our understanding on GA/ABA antagonism which is important to know how plants cope with changing environments. Thus, deepwater rice was considered as a model plant to study stem elongation and basic aspects of plant growth (Kende et al. 1998).

In addition, Rayada rice seems to be an excellent material for natural stress memory phenomenon study (Chinnusamy and Zhu 2009) as it always passes through multiple stresses (flooding, cold, sometimes drought) before completion of their life cycle. Similarly, lack of dormancy of Rayada may be also an adaptive trait and the genetic basis of such adaptive feature is the key question of both fundamental and evolutionary biology. But both physiological and molecular bases of this trait of Rayadas are completely unknown, so systemic study on the feature of lack of dormancy of Rayada rice could be an interesting topic of scientific interest.

Although both IRRI and BRRI dimmed their focus on deepwater varietal improvement programme, moreover, kept it out of their priority list, but the prime area of research of both of the institutes are development of abiotic stress tolerant rice, mainly from drought, submergence and salinity (Nasiruddin and Hassan 2009). After reviewing and analysing physiological specialty of Rayadas, we are convinced with their special traits that could be certainly used as excellent resources for future crop improvement programme as well as basic scientific studies. However, direct use of Rayadas as breeding material would be more unlikely as it also possesses several undesired traits such as longer life cycle, strong photoperiod sensitivity etc. Moreover, molecular insights of most of these traits are not yet clearly understood. So more detailed research is needed to characterize its special traits and then mine its useful genes.

## Conclusion

Rayada is such a primitive deepwater rice ecotype completely endemic to certain area of Bangladesh that can offer many traits and genetic resources that are badly needed in rice breeding for stress tolerance. Moreover, it could be an excellent material for basic scientific studies like natural stress memory phenomenon, indica-japonica differentiation as well as natural adaptive features like lack of dormancy. We expect Rayada rice will become a new focus of rice germplasm research and think the time is mature to do so with the rapid advances in genome research weaponry.

## Abbreviations

DWR: Deepwater rice; IRRI: International rice research institute; BRRI: Bangladesh rice research institute; EUI: Elongated uppermost internode; ERF: Ethylene response factor; SNP: Single nucleotide polymorphism; RLD: Root length density; BVP: Basic vegetative phase; PSP: Photoperiod sensitive phase; GWAS: Genome-wide association study; QTL: Quantitative trait loci.

## Competing interests

The authors declare that they have no competing interests.

## Authors’ contributions

ANMRBR drafted the manuscript and performed the experiments. JZ conceived of the ideas of the article and revised the manuscript. Both of the authors read and approved the final manuscript.
